# Adeno-associated virus receptor complexes and implications for adeno-associated virus immune neutralization

**DOI:** 10.3389/fmicb.2023.1116896

**Published:** 2023-02-10

**Authors:** Edward E. Large, Michael S. Chapman

**Affiliations:** Department of Biochemistry, University of Missouri, Columbia, MO, United States

**Keywords:** adeno-associated virus, AAVR, entry, antibody, gene therapy, structure, electron microscopy, cryo-EM

## Abstract

Adeno-associated viruses (AAV) are among the foremost vectors for *in vivo* gene therapy. A number of monoclonal antibodies against several serotypes of AAV have previously been prepared. Many are neutralizing, and the predominant mechanisms have been reported as the inhibition of binding to extracellular glycan receptors or interference with some post-entry step. The identification of a protein receptor and recent structural characterization of its interactions with AAV compel reconsideration of this tenet. AAVs can be divided into two families based on which domain of the receptor is strongly bound. Neighboring domains, unseen in the high-resolution electron microscopy structures have now been located by electron tomography, pointing away from the virus. The epitopes of neutralizing antibodies, previously characterized, are now compared to the distinct protein receptor footprints of the two families of AAV. Comparative structural analysis suggests that antibody interference with protein receptor binding might be the more prevalent mechanism than interference with glycan attachment. Limited competitive binding assays give some support to the hypothesis that inhibition of binding to the protein receptor has been an overlooked mechanism of neutralization. More extensive testing is warranted.

## 1. Introduction

Adeno-associated virus (AAV) is a small parvovirus that has garnered attention as a gene therapy vector. The virus naturally infects humans and is considered non-pathogenic, in part, because AAV cannot replicate absent a helper virus with its own often-dominant pathology (Salo and Mayor, 1979). The underlying biology of AAV is also well understood and this knowledge has been used to create recombinant AAV (rAAV), which has lower antigenicity and less severe side effects than other gene therapy vectors (Berns and Giraud, 1996; Carter and Samulski, 2000; Carter et al., 2008). AAV has become the vector of choice in the first gene therapies approved by the FDA to treat RPE65-associated retinal dystrophy, spinal muscular atrophy, and hemophilia B (Von Drygalski et al., 2019; Mendell et al., 2021).

AAV is a 4.7 kb, non-enveloped, single-stranded DNA virus of the family *Parvoviridae*, genus Dependovirus (Penzes et al., 2020). The AAV genome contains two major gene regions consisting of an upstream *rep* gene and a downstream *cap* gene. The *rep* gene produces Rep proteins from a single ORF (Open Reading Frame) *via* alternative splicing and the Rep proteins have important roles in AAV packaging, replication and integration. The cap gene produces three viral capsid proteins (VP1, VP2, and VP3) *via* alternate start codons and VP1-VP3 are present as a stochastic distribution in particles, averaging a molar ratio of 1:1:10, respectively (Wörner et al., 2021). At least three additional alternate ORFs have been discovered in the *cap* region and these encode the proteins AAP, MAAP, and X; all of which have no known roles associated with capsid structure (Sonntag et al., 2011; Naumer et al., 2012; Cao et al., 2014, 2015; Stutika et al., 2016; Earley et al., 2017; Tse et al., 2018; Maurer et al., 2019; Ogden et al., 2019).

A number of AAV serotypes have been isolated from humans and nonhuman primates (Gao et al., 2004). AAVs can be grouped into eight clades and structures exist for all commonly used capsids. There are 13 major primate serotypes grouped into six clades (A–F) with one unique distantly related group (AAV4, 11 and 12) and a single distantly related isolate (AAV5). Each serotype has a unique tropism that depends on the capsid sequence and topology. The eight clades each contain at least one antigenically distinct serotype.

The exposure of AAV to the immune system, either *via* natural means or *via* recombinant vectors, creates an antibody response. Viral antigenic sites (epitopes) can be recognized by antibodies and many people are seropositive to commonly used serotypes (Erles et al., 1999). The presence of neutralizing antibodies (nAbs) limits transduction by rAAV. This is a potential challenge for those exposed naturally to AAV, which elicits neutralizing antibodies with a single dose, or repeat doses of gene therapy (Moskalenko et al., 2000; Li et al., 2009; Weber, 2021). It is important to understand how AAV is recognized and neutralized so that strategies for evading the neutralization of vectors can be devised.

The current understanding of AAV neutralization by antibodies comes primarily from virological and structural studies of monoclonal antibodies (mAb). Against 9 serotypes, a small library of 25 mAb has been raised, many neutralizing (Wobus et al., 2000; Kuck et al., 2007; Gurda et al., 2012; Harbison et al., 2012), using the sera of lab animals challenged with AAV capsid proteins. Cryo-electron microscopy (EM) has defined conformational epitopes for 13 at varying resolution (Gurda et al., 2012; McCraw et al., 2012; Gurda et al., 2013; Tseng et al., 2015; Bennett et al., 2018; Giles et al., 2018; Jose et al., 2019). Mechanisms of neutralization have been thought to involve primarily inhibition of glycan-binding or a post-entry step (Gurda et al., 2013; Tseng and Agbandje-McKenna, 2014; Tseng et al., 2015).

In this review, mounting evidence (that is circumstantial, but strong) is presented of a need to reappraise the likely mechanisms of neutralization. A changed perspective is prompted by the new understanding of the role of cellular glycans as attachment factors rather than classical receptors (Meyer et al., 2019; Meyer and Chapman, 2022). AAV attachment factors, for the purposes of this review, are external cellular factors with low-specificity interactions that lead to the accumulation of AAV virions on the cell surface, whereas co-receptors, while insufficient alone, enhance AAV cellular entry. The discovery of AAVR (AAV Receptor) has led to an increasing number of structural studies that are revealing overlap between neutralizing epitopes and the binding sites of the AAVR membrane protein receptor that is essential for productive cellular entry by most serotypes (Meyer et al., 2019; Silveria et al., 2020).

## 2. AAV glycan attachment factors

**Figure 1:**
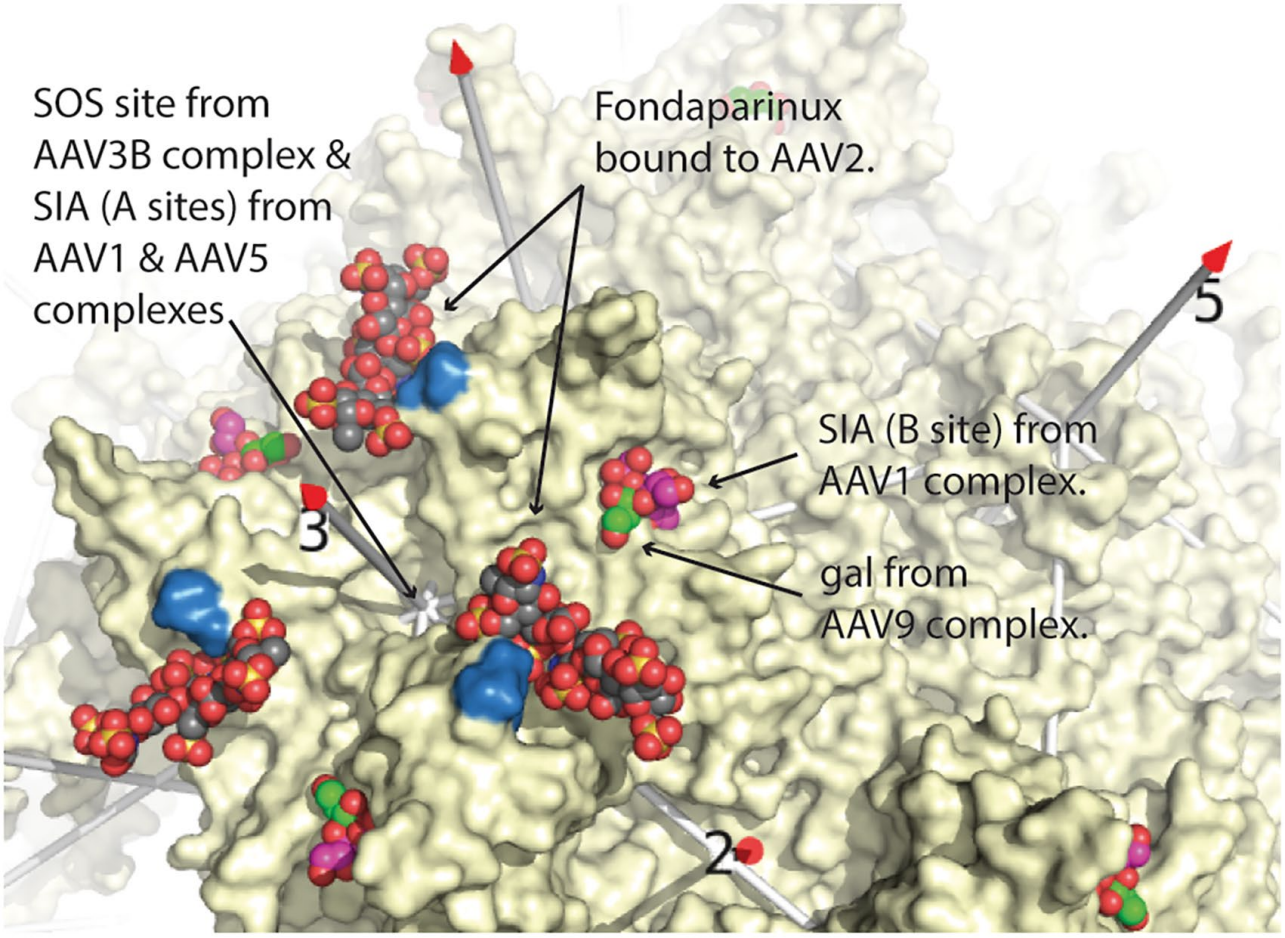
Comparison of glycan attachment sites overlaid on the structure of AAV2, from Stagg et al. (2022). Equivalent sites are shown surrounding one of the three-fold axes of symmetry of AAV. Arrows point to space-filling representations of fondaparinux, a heparin analog from the AAV2 cryo-EM complex (Xie et al., 2017), sialic acid (SIA, magenta carbons) from the AAV1 crystal structure (Huang et al., 2016), and galactose (gal; green carbons) from the cryo-EM AAV9 structure (Penzes et al., 2021), the latter two overlapping. Two arginines (585 & 588), important in AAV2 heparan-binding, are colored blue (Kern et al., 2003). Additional glycan attachment interactions for AAV1, AAV3B, and AAV5 have also been inferred through crystallography or mutational studies (Lerch and Chapman, 2012; Afione et al., 2015; Huang et al., 2016). Structures were overlaid by superimposing the AAV VP3 capsid proteins using the SSM sequence-structure alignment as implemented in Pymol (DeLano, 2002; Krissinel and Henrick, 2004; The PyMOL Molecular Graphics System, 2.0, 2015), and then expanding the icosahedral symmetry.

Starting with the requirement of AAV2 for heparan sulfate proteoglycan in mediating attachment and infection (Summerford and Samulski, 1998), extracellular glycans of several types were implicated as receptors for different groups of AAV serotypes (Agbandje-McKenna and Kleinschmidt, 2011; Huang et al., 2014). In the 1998 paper, strong evidence for the glycan in attachment was presented, while the implication as a receptor was a plausible inference that took hold throughout the community (Summerford and Samulski, 1998). Two glycans (or the interacting fragments thereof), heparan sulfate (HS) and sialic acid (SIA) are negatively charged whereas the third, galactose, is uncharged. HS is known to be important for the cellular attachment of serotypes AAV2, 3B, 6 and 13 (Summerford and Samulski, 1998; Handa et al., 2000; Halbert et al., 2001; Schmidt et al., 2008). AAV1, 5, and 6 use SIA and AAV9 uses galactose to attach to cells. Glycans with a terminal sialic acid (SIA) were found to be important for AAV1, 4, 5, and 6 (Kaludov et al., 2001; Wu et al., 2006), while glycans with a terminal galactose were implicated in AAV9 cellular entry. Structures for AAV1 and AAV5 complexed with SIA and, more recently of galactose bound to AAV9 (Penzes et al., 2021) have revealed that HS, SIA, and galactose glycan attachment sites are mostly distinct (Figure 1).

Additional surprises came. Systematic studies, comparing glycan-binding using libraries of different heparinoids or SIA, revealed unexpectedly low specificity for the glycans that had previously been implicated by cellular transduction observations (Wu et al., 2006; Zhang et al., 2013; Mietzsch et al., 2014; Xie et al., 2017). Furthermore, several heparinoids bound with greater avidity than the HS that had been implicated in cellular infection (Summerford and Samulski, 1998). Then, evidence emerged that glycan-binding and cell-transduction could be modulated by chimeric grafting into an AAV2 or AAV3 background, glycan binding-site amino acids from AAV2, AAV3, or AAV9 (Lerch and Chapman, 2012; Shen et al., 2013). Recently, evidence has been mounting that HS-binding might be an adaptation to laboratory propagation in cultured cells: AAV2 has higher HS affinity and lower *in vivo* liver tropism, relative to natural ancestors identified by DNA sequences from primary cell cultures (Cabanes-Creus et al., 2020). The ancestors are not yet as well characterized, but establish that HS is not an absolute-requirement for AAV2-like strains (Cabanes-Creus et al., 2020). Even before these latest findings, the evidence contrasted with expectations of a classical receptor and were suggestive of an attachment-only role that was mediated by non-specific bulk interactions between AAV and various glycans on the cell surface.

## 3. Co-receptors

In addition to glycans, several cell surface membrane proteins were identified as entry determinants. Considering their less critical role, they were termed “co-receptors” or “secondary receptors,” to distinguish them from glycan “primary” receptors. Seven different co-receptors were identified for one or more of the different serotypes (Goldman and Wilson, 1995; Qing et al., 1999; Summerford et al., 1999; Di Pasquale et al., 2003; Kashiwakura et al., 2005; Akache et al., 2006; Asokan et al., 2006; Weller et al., 2010). These included integrins, laminin, platelet-derived growth factor receptor (PDGFR), hepatocyte growth factor receptor (HGFR/c-Met), and fibroblast growth factor receptor (FGFR). Through attempts at structural characterization, evidence for physical interactions between AAV and expressed domains from several co-receptors remained elusive, while siRNA inhibition of the growth factor receptors had little or no impact (unpublished). With hindsight, we now see that in three independent genome-wide screens of host cell knockout libraries, none of the previously implicated co-receptors were confirmed as having appreciable impact upon transduction (Pillay et al., 2016, 2017; Dudek et al., 2020a; Meisen et al., 2020). Directly targeted knock-out of FGFR1 or c-Met by CRISPR or TALENs had no or small impact upon AAV2 transduction (Pillay et al., 2016). Cautions must include the insensitivity of such methods to subtle accessory effects, the challenge of proving a negative, and reliance on screening data because not all previously proposed co-receptors have been re-examined by targeted knock-out. Co-receptors may still play important yet unidentified roles in entry, but co-receptors no longer appear to have the central role once thought.

From today’s perspective, it seems that many of the co-receptors play at most minor accessory roles. It is possible that some may have impacted reporter transgene transduction levels, not through direct physical interactions with the virus, which have never been established, but perhaps by modulating the state of the cell, particularly for the co-receptors whose native functions are as growth factor receptors (Pillay et al., 2016; Meyer and Chapman, 2022).

## 4. AAV antibody complexes

The mapping of antibody epitopes to the AAV structure started with peptide and amino acid substitution scanning (Moskalenko et al., 2000; Wobus et al., 2000; Lochrie et al., 2006), but latterly has been primarily through cryo-EM (Gurda et al., 2012; McCraw et al., 2012; Gurda et al., 2013; Tseng et al., 2015; Bennett et al., 2018; Giles et al., 2018; Jose et al., 2019). Pragmatically, it is easier to work with purified samples, and so our understanding of what might happen in an *in vivo* human polyclonal response is by extrapolation from a subset of the 25 mAb raised to 9 serotypes, many of which are neutralizing (Wobus et al., 2000; Kuck et al., 2007; Gurda et al., 2012; Harbison et al., 2012). The neutralizing mAb available are all from animal models. Human mAb have been isolated, but all to-date are binding, not neutralizing, and attempts at structural epitope mapping have not yet succeeded (Giles et al., 2020). Notwithstanding that studies of mAb-binding might not be fully representative of polyclonal neutralization mechanisms (Dudek et al., 2020a), this study focuses on the mAb for which epitopes can be mapped structurally. The first structure was of the complex of AAV2 with Fab’ fragments of strongly neutralizing mAb A20 (McCraw et al., 2012). Subsequent studies were mostly from Mavis Agbandje-McKenna and colleagues who have provided comparative reviews (Tseng and Agbandje-McKenna, 2014; Emmanuel et al., 2021), while an updated table of AAV-mAb structures is provided by Stagg et al. (2022).

Lessons from other viruses are that nAb can neutralize directly through a variety of mechanisms, including inhibition of receptor-binding, aggregation, structural strain, and inhibition of required conformational change (Smith and Moser, 1997; Kwong et al., 1998; Smith, 2001). By the time that the structures of mAb-AAV complexes were solved, glycan binding sites had been determined. In the days where glycans were considered primary receptors, these were believed to be among the regions of the AAV surface most critical to viral entry. With glycans still regarded as receptors, and not yet demoted to attachment factors, it would be natural to assume that cellular attachment assays could be used as a proxy in inferring nAb-inhibition of receptor-mediated entry (Wobus et al., 2000). Mechanisms of neutralization were deemed to be “postbinding” or “post-entry” for several antibodies where little impact on cellular attachment was measurable (Tseng and Agbandje-McKenna, 2014; Silveria et al., 2020; Emmanuel et al., 2021). It only later became clear that attachment to the cell surface was dominated by glycan interactions, and not significantly dependent on the membrane protein interactions that were required for productive cell entry (Pillay et al., 2016). Conclusions were conditioned by the prevailing understanding at the time, and we need to be open to reinterpretation of the experimental data.

As we reanalyze possible mechanisms of neutralization in light of new receptor complex structures (below), it should be remembered that most of the structures are at low resolution and among the more approximate of AAV structures. The lone exceptions are the structures of HL2476, solved at 3.1 Å resolution at which most side chains should be visible (Jose et al., 2019), and PAV9.1 at 4.1 Å resolution at which backbone should be clear (Giles et al., 2018). The majority of structures were solved before the technical “resolution revolution” in cryo-EM (Cheng, 2015), at resolutions from 7 to 23 Å resolution. In this range, the details depend on the fidelity of a homology-based prediction of antibody domain structures, fit into an EM-based envelope of decreasingly defined outline. The implications of these structures will be re-examined below, in light of new structures of receptor-complexes. This could have been pursued more rigorously had the structures and underlying EM maps been deposited in public databases (Berman et al., 2000; Lawson et al., 2011). However, with one exception (McCraw et al., 2012), the prevailing standards of deposition on publication had not been enforced. Thus, for the most part, potential conflict between the binding of antibodies and receptors can be assessed only indirectly through lists of contact residues published according to the authors’ interpretations.

## 5. AAV receptor complexes

Recapitulating, the glycans, previously described as “primary receptors,” did not have the specificity that would be expected of a classical cell entry receptor. Attempts to characterize physical complexes between AAV and co-receptors had failed, noting both that it was only a subset of the previously identified co-receptors that had been targeted and that it would always be difficult to prove beyond a doubt the negative that none of the identified co-receptors were critical. Nevertheless, it seemed likely that host factors key to successful cell entry had not been identified.

This motivated relatively unbiased approach using genome-wide screening and the first proteinaceous AAV receptor necessary for cellular entry, adeno-associated virus receptor (AAVR), was identified in three independent genome-wide screens (Pillay et al., 2016; Dudek et al., 2020a; Meisen et al., 2020). Success had come first using a gene trap library (Pillay et al., 2016), with subsequent screens using CRISPR knock-out libraries yielding substantially similar results (Dudek et al., 2020a; Meisen et al., 2020) and agreement on the principle suspects (Meyer and Chapman, 2022). From these screens, it was evident that the hitherto uncharacterized membrane protein, encoded by the KIAA0319L gene, had much more significant impact upon AAV2 transduction than heparan sulfate proteoglycan or two previously identified co-receptors (Pillay et al., 2016). *In vivo*, AAVR knock-outs ablated transduction in mice, while, *in vitro*, AAVR was important for all cell lines and most AAV serotypes tested (Pillay et al., 2016). Intriguingly, AAVR was found to colocalize with trans-Golgi network (TGN) components (Pillay et al., 2016), as does AAV2 when trafficking from the plasma membrane through endosome compartments to the Golgi.

AAV entry was initially characterized as following a clathrin-mediated endocytic pathway (Duan et al., 1999; Bartlett et al., 2000). Subsequent studies, by contrast, implicated clathrin-independent carriers (CLIC) in AAV endocytosis (Nonnenmacher and Weber, 2011). There might be several pathways that lead AAV virions to the Golgi (Nonnenmacher et al., 2015) and possibly differences between cell lines (Nonnenmacher et al., 2015; Riyad and Weber, 2021). AAVR^KO^ rescue experiments using AAVR with alternate C-terminal signal peptides indicate that intracellular trafficking of AAV2 through the trans-Golgi network (TGN) is not absolutely required, but greatly enhances transduction efficiency (Pillay et al., 2016).

**Figure 2:**
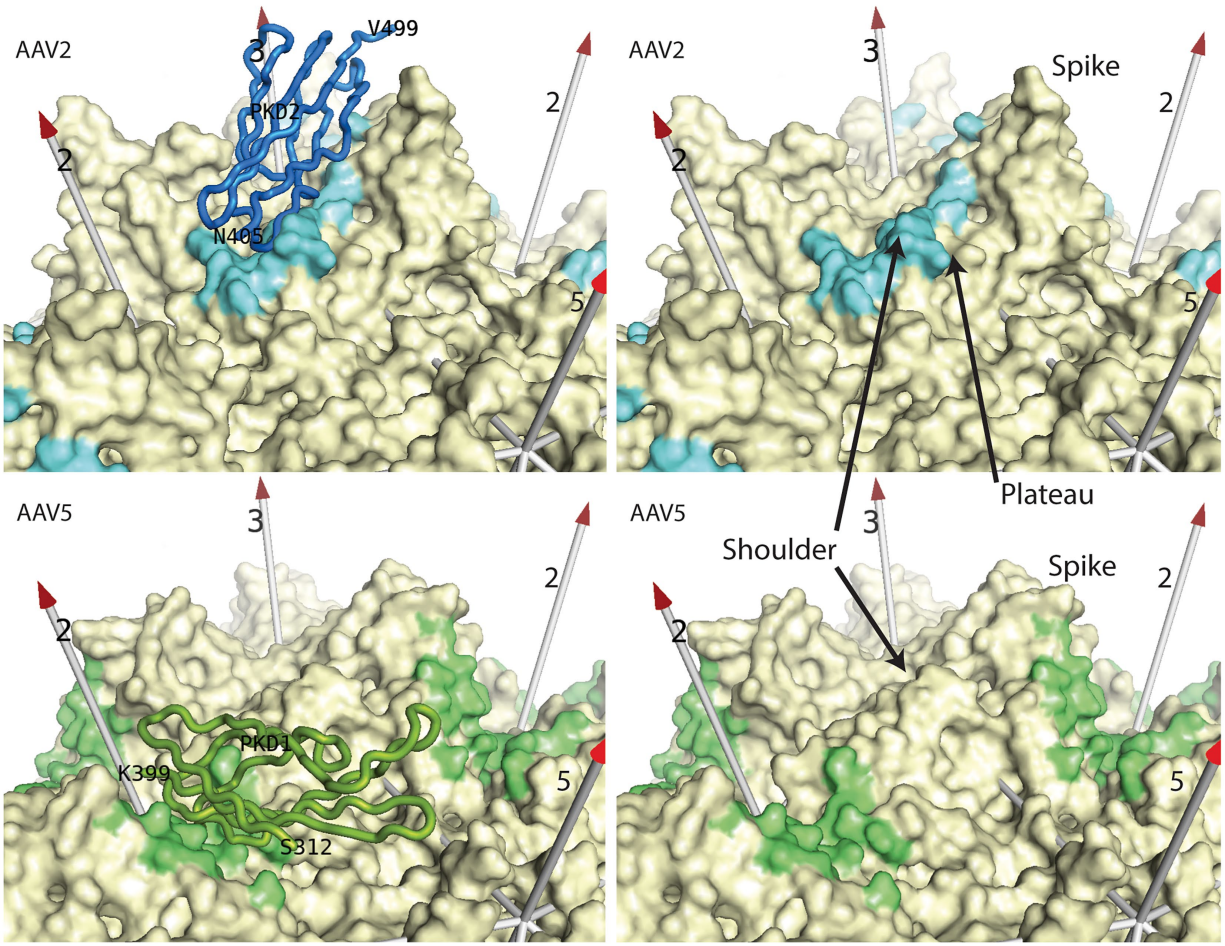
AAV receptor binding sites. Most AAV serotypes bind primarily to the PKD2 domain (blue) of AAVR as seen in the 2.4 Å cryo-EM structure of the complex (Meyer et al., 2019). A smaller clade binds to the PKD1 domain (green) as exemplified in the 2.5 Å cryo-EM structure (Silveria et al., 2020). In the right panels, the AAVR domains have been removed to reveal the contact footprints (4.5 Å cut-off) for all of the binding surfaces that are related by the viral symmetry axes (numbered arrows). Prominent features of AAV’s surface are annotated.

Cryo-EM structures are revealing the architecture of AAV-AAVR interactions. AAVR is a glycoprotein (N-linked and O-linked) comprised of multiple domains from N- to C-terminus: a “motif with eight cysteines” (MANEC) domain, five immunoglobulin-like polycystic kidney disease (PKD) domains (PKD1-5), and a C-terminal transmembrane region. PKD1-5 were identified as important for AAV transduction (Pillay et al., 2016; Pillay and Carette, 2017). AAV5 requires PKD1, whereas AAV2 primarily uses PKD2 with contributions from PKD1 (Pillay et al., 2017). Several cryo-EM structures of AAV-AAVR complexes are published using constructs containing either two domains (PKD12) or all five PKD domains (PKD1-5) with similar results (Meyer et al., 2019; Zhang et al., 2019; Silveria et al., 2020; Xu et al., 2022). The majority of structures contain serotypes bound to PKD2 (AAV1, AAV2 and AAV9) with the PKD2 N-terminus observed starting near the AAV 2-fold axis with β-strands looping back and forth until the domain’s C-terminus near the viral 3-fold axis (Figure 2; Meyer et al., 2019; Zhang et al., 2019; Xu et al., 2022). Two structural studies of AAV5 resolve the N-terminus of PKD1 near the 5-fold axis with the domain descending toward the C-terminus near the 2-fold axis (Figure 2; Zhang et al., 2019; Silveria et al., 2020). Another AAV5 clade member, AAVGo.1, also binds to human PKD1 albeit with a ~ 1 Å rotation of the receptor when compared to AAV5 (Large et al., 2022). Therefore, two structural classes of PKD binders are currently known: AAV5-like AAVs that bind PKD1 at one site with some rotational variation in domain docking and the larger set of other AAVs that binds PKD2 at a different site, but with even higher uniformity of orientation.

**Figure 3:**
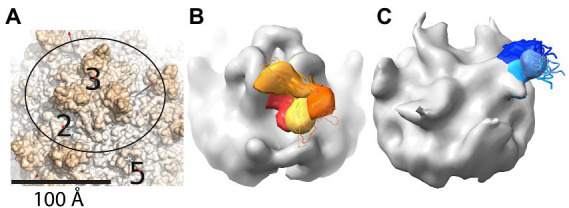
Cryo-ET reveals variable locations for neighboring PKD domains that are not strongly bound to AAV. **(A)** The region shown in panels **(B,C)** is outlined on part of the surface of the AAV2 crystal structure (PDB ID 1LP3) (Xie et al., 2002). Spikes are seen surrounding a 3-fold axis, with neighboring 2- and 5-fold axes shown for reference. **(B)** Cryo-ET subtomograms for an AAV2-PKD12 complex have been averaged within four classes that are superimposed with different colors (yellow through red) for the PKD1 domain that is in variable orientation. Regions in common between the classes are colored grey, and comparison with panel **(A)** shows the added mass of PKD2 running up from near the 2-fold to above the 3-fold spike. **(C)** Cryo-ET subtomogram class averages for AAV5-PKD12 that have been similarly represented to show the variable orientations of PKD2 (shades of blue) emanating from a symmetry-related 2-fold axis behind the spike. PKD1 is bound in a single configuration, adding to the grey volume (*cf.* panel **A**) between the 5- and 2-fold axes. Panels are taken with permission from Hu et al. (2022).

Single particle (conventional) cryo-EM was unable to explain the ancillary role of PKD1 in AAV2 transduction (Pillay et al., 2016, 2017), because, in spite of its presence in the samples, it was not resolved in any of the high-resolution structures. At first, it was suspected that PKD1 might lie in a position on AAV2 similar to the location seen on AAV5 (Zhang et al., 2019; Silveria et al., 2020) but, without strong binding, too disordered to resolve at high resolution. This was ruled out, because the linker between PKD1 and PKD2 domains is 5 amino acids and too short to bridge the 19 Å gap when PKD1 and PKD2 were superimposed from their respective AAV5 and AAV2 complex structures (Silveria et al., 2020). Cryo-Electron Tomography (ET) is more suitable for (low resolution) characterization of highly heterogeneous conformations, because 3D information is available for every particle, facilitating the separation into different classes of conformer and then intra-class averaging of sub-tomograms (Hu et al., 2022). A recent cryo-ET study of AAV2 found PKD2 bound as it had been in the cryo-EM, but now with PKD1 radiating outward, connected by its flexible linker in several alternate orientations (Figure 3). None corresponded to the AAV5 location of PKD1, and none had more than minimal contact with the AAV2 surface (Hu et al., 2022). Corresponding cryo-ET of an AAV5 complex revealed PKD2 linked to the AAV5-bound PKD1, again pointing away from the virus, now in three orientations (Hu et al., 2022). So, there is not yet a clear and unique explanation of the accessory role of PKD1 in AAV2-AAVR binding. One of the AAV2 PKD1 orientations makes modest contact with the capsid, but it is equally plausible that the presence of PKD1 has an indirect effect by stabilizing neighboring PKD2, or that AAV2-binding is impacted by a yet-uncharacterized dynamic equilibrium of oligomeric AAVR assemblies that is PKD1-dependent.

## 6. Potential competition: Antibodies vs. glycan attachment and receptor binding

Potential inhibition of glycan-attachment by neutralizing antibodies can be rationalized, in several cases, through overlap of the experimentally determined antibody epitopes with glycan-interacting amino acids. Several caveats need to be considered. Firstly, glycan-interactions have been determined using small saccharides (sialic acid, galactose, fondaparinux, etc.). However, the virologically relevant interaction is with a much larger polysaccharide. We must allow for the greater potential for antibody overlap of a larger carbohydrate, but can only guess at the proximity required to block attachment. Secondly, the 3D antibody structures are not publicly available, so we must extrapolate from lists of epitope residues to conjecture where antibody and glycan might conflict above the viral surface. Finally, the resolutions of the antibody complexes vary widely, so there may be up to near-nanometer uncertainty in some of the epitope boundaries. In summary, there are many cases where structure can be used to rationalize the possibility that there might be interference with glycan attachment, but there is significant uncertainty in most of these cases about whether the binding sites really overlap and have the potential for competitive inhibition.

**Figure 4:**
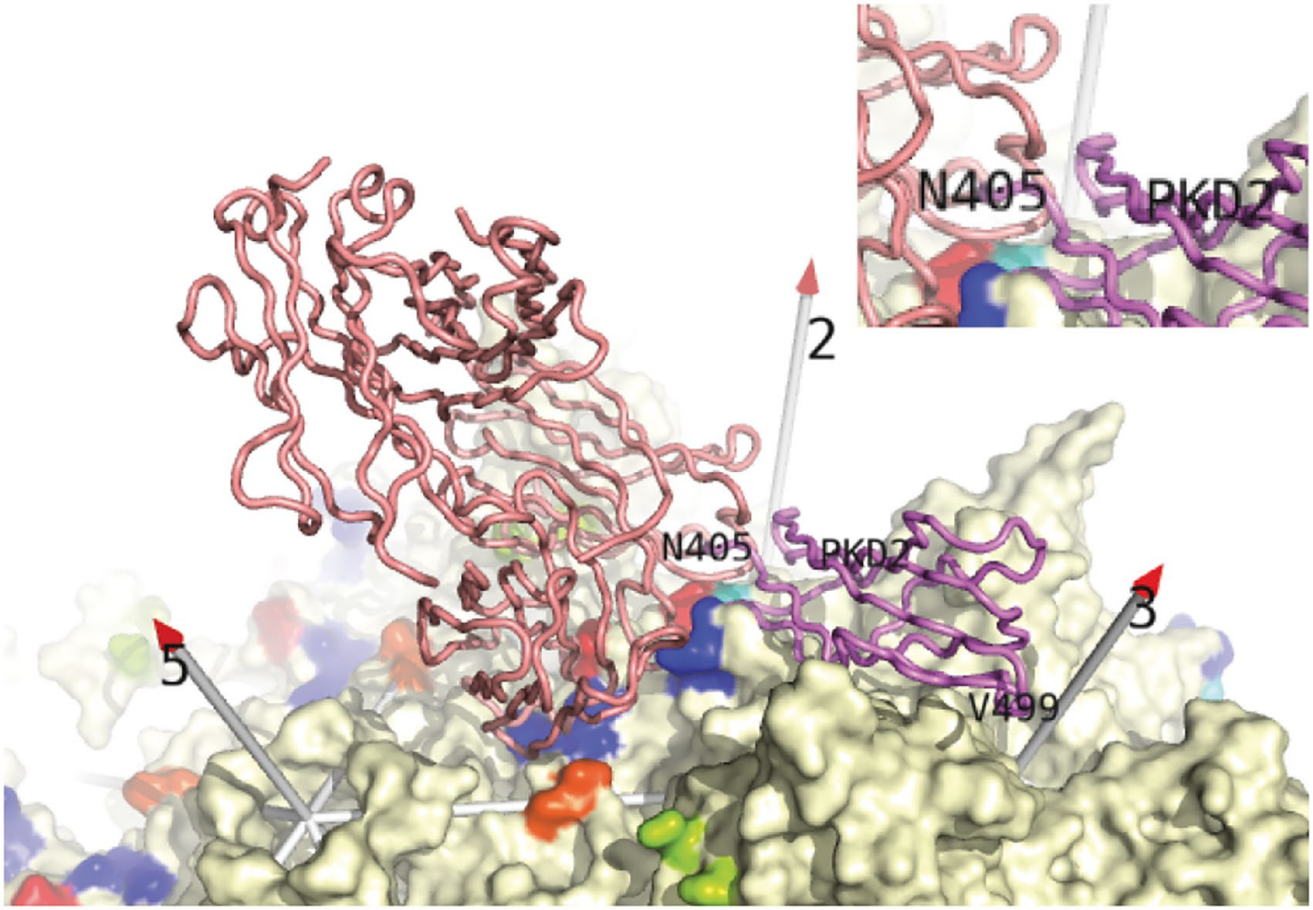
Conflict between neutralizing mAb A20 and the AAVR receptor from Stagg et al. (2022). Backbone traces of AAVR PKD2 domain (violet) and the A20 Fab’ structure (salmon) from the structures of AAV2 complexes are overlaid on the surface of the virus (McCraw et al., 2012; Xie et al., 2017; Meyer et al., 2019). Conflict, highlighted in the inset, occurs between the N-terminal residues of PKD2 and the CDR loops of mAb A20 above the spur extending from AAV2’s 3-fold spike. PKD1 was not seen in the single-particle cryo-EM structures (Meyer et al., 2019; Zhang et al., 2019), but was seen at low resolution in recent cryo-electron tomography (Cryo-ET) (Hu et al., 2022). PKD1 is in four orientations extending beyond PKD2 Asn_405_, adding extensively to the overlap with mAb A20. A20 is the only antibody whose complex with AAV is deposited in the structural databases. For structures to which we do not have access (Figure 5), one may still highlight AAV residues reported to be in contact. (Those within 4 Å of A20 are rainbow colored here). Then one could judge how each antibody would be placed to make the identified contacts, and whether there would likely be receptor-overlap.

**Figure 5:**
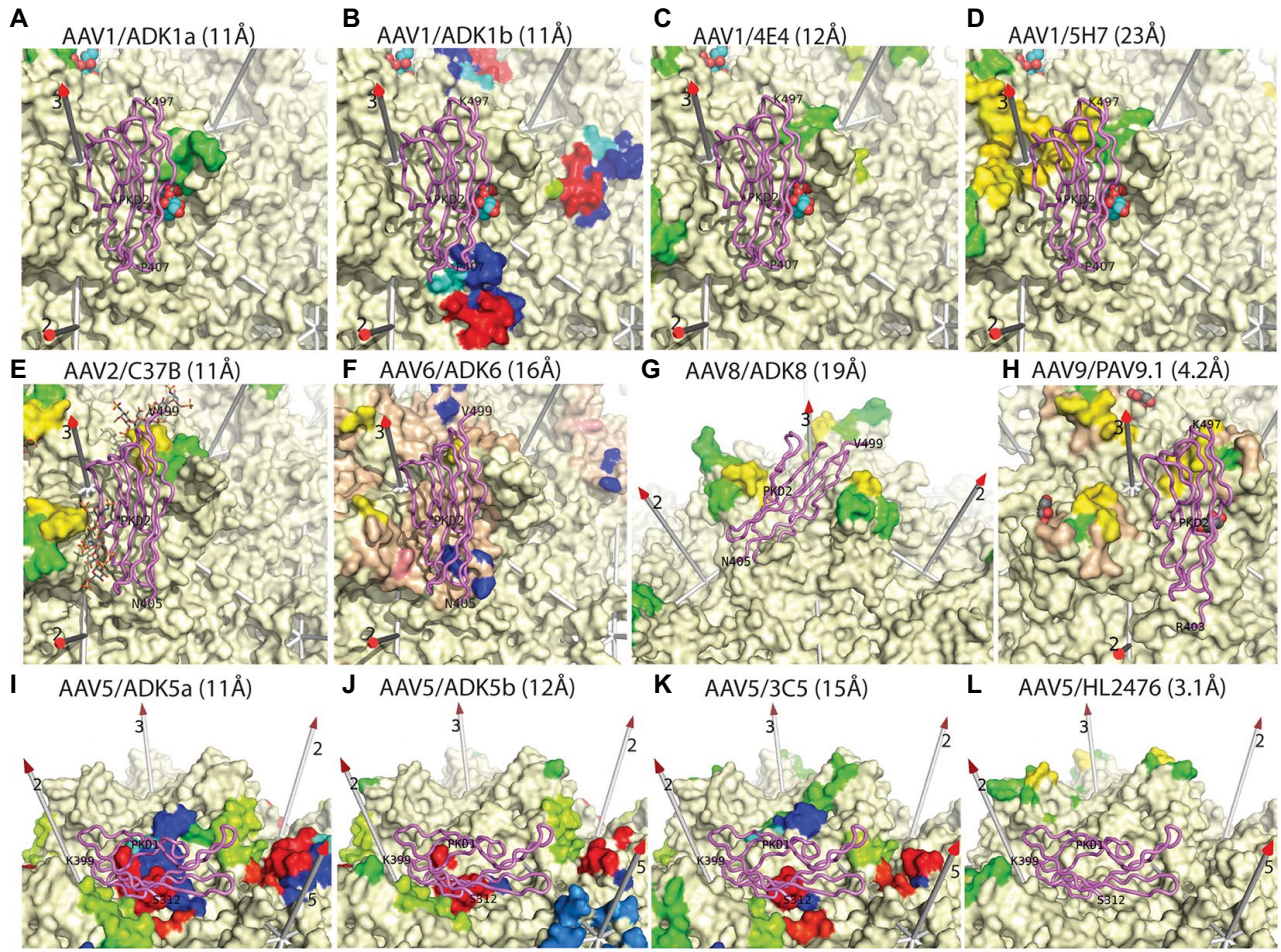
Epitopes of additional anti-AAV monoclonal antibodies. This figure is similar to that in Stagg et al. (2022), but updated to include the most recent structures of AAV-receptor complexes (violet backbone traces), with PKD1 resolved for AAV5 and PKD2 resolved for AAVR complexes with other serotypes (Meyer et al., 2019; Zhang et al., 2019; Silveria et al., 2020; Xu et al., 2022). Surface residues of several AAV serotypes are rainbow-colored according to residue number if reported to be in contact with the mAb indicated in the panel label, as inferred from cryo-EM structures at the noted resolutions (Xie et al., 2002, 2011; Nam et al., 2007; DiMattia et al., 2012; Gurda et al., 2012, 2013; Tseng et al., 2015; Bennett et al., 2018; Giles et al., 2018; Jose et al., 2019; Zhang et al., 2019; Silveria et al., 2020). Other parts of the surface are cream colored, except as noted below. Wheat-colored regions (panels **F,G**) were reported as occluded by antibody-binding (Bennett et al., 2018; Giles et al., 2018). Known glycan attachment sites are indicated as overlaid spheres for sialic acid or stick-model for fondaparinux (panels **A–I**) (Huang et al., 2016; Xie et al., 2017), while amino acids implicated in glycan-binding by mutational analysis are salmon-colored in panel **F** (Xie et al., 2011; Huang et al., 2016). Conclusions to be drawn from this comparison are: (1) many neutralizing antibodies occlude glycan attachment, but not all; (2) the binding of all nAb conflicts with the serotype-relevant AAVR domain. In most cases, PKD1 (AAV5) or PKD2 (other serotypes) lies directly over the neutralizing epitope, but for HL2476 (panel **L**) conflict is with unseen inter-domain linker.

Strongly neutralizing mAb A20 binds on the plateau below the 3-fold spikes of AAV2, in the direction of the 5-fold (McCraw et al., 2012; Figure 4). There is no overlap with the binding of heparan sulfate in the valley between neighboring 3-fold spikes (McCraw et al., 2012), consistent with the lack of inhibition of cell attachment (Wobus et al., 2000). Let us now compare the antibody complexes for which atomic structures have not been released, but where epitope amino acids have been identified (Figure 5). Neutralizing anti-AAV2 C37B binds to the 3-fold spike, proximal to the heparin site (Figure 5E; Gurda et al., 2013), possibly accounting for the observed inhibition of cell attachment (Wobus et al., 2000), although there is not direct overlap. Neutralizing anti-AAV1 mAb, ADK1a, binds immediately proximal to the sialic acid (SIA) binding site, while ADK1b, 4E4, and 5H7 bind to surrounding regions of the surface, several residues away (≥7 Å), in different directions (Figures 5A–C; Gurda et al., 2013; Mietzsch et al., 2015; Tseng et al., 2015; Huang et al., 2016). For ADK1a, this proximity, together with the AKD1a-escape of SIA-null mutants, suggested that the attachment-interference was the mechanism of neutralization (Huang et al., 2016; Emmanuel et al., 2021). Neutralizing mAbs 4E4 and 5H7, but not ADK1b, inhibit cell attachment (Harbison et al., 2012; Emmanuel et al., 2021). Based on overlap of the epitope and AAV6 glycan-binding sites (Figure 5F), it was predicted that ADK6 blocked glycan attachment, in the absence of empirical data (Bennett et al., 2018). For AAV9, mAb PAV9.1 binds at the top of the 3-fold spike, above the galactose attachment site at its base (Figure 5H; Bell et al., 2012; Giles et al., 2018), not unlike some of the anti-AAV1 mAb, such as ADK1a. Finally, let us consider the anti-AAV5 mAbs shown in Figures 5I–L. Two SIA sites have been identified, of which only the A-site (in the 3-fold depression) has been associated with SIA-dependent cell transduction (Afione et al., 2015) and is closer to the epitopes. HL2476 binds to the tip of the spikes, close enough at 6 Å, it was proposed, to partially occlude glycan-attachment (Figure 5L; Jose et al., 2019). Antibodies 3C5, ADK5a, and ADK5b bind progressively further away, with glycan-occlusion seeming increasingly unlikely, though it was only ADK5b that had previously been reported as neutralizing (Harbison et al., 2012; Tseng et al., 2015) (but see below).

In summary, among the 13 complexes that can be evaluated, ADK6 is the sole example where the epitope overlaps directly with the glycan-binding site. For ADK1a and C37B, the epitope is immediately proximal to glycan attachment, so interference is reasonable to postulate. For half of the remaining complexes, there is not a strong case, but one should not completely exclude the possibility that mAb-binding in the general vicinity could impact glycan attachment, particularly because only a small part of the glycan has been visualized structurally. However, it would be wise to be open to other possible mechanisms of neutralization.

Cryo-EM structures of complexes of AAV2 with extracellular fragments of AAVR (Meyer et al., 2019; Zhang et al., 2019) allowed assessment of potential conflict between antibody and (protein) receptor binding. Overlap was not recognized in one of the studies (Zhang et al., 2019), but when the symmetry of the virus is accounted for, it is clear that mAb A20 occludes AAVR PKD2 bound at the overlapping site (Figure 4; Meyer et al., 2019; Meyer and Chapman, 2022). For students of more exhaustively studied virus-antibody interactions, it would be no surprise that antibodies can neutralize virus capsids through a wide variety of mechanisms (Sherman et al., 2020), including inhibition of receptor-binding. However, at the time that the first AAV nAb were characterized (Wobus et al., 2000), glycans were considered to be the entry receptors and antibodies were categorized as attachment-inhibiting or not. Those failing to inhibit attachment were proposed to have post-binding mechanisms of neutralization (Wobus et al., 2000). It was not anticipated that mAb A20, commonly characterized as a “post-entry” neutralizer (Tseng and Agbandje-McKenna, 2014; Silveria et al., 2020; Emmanuel et al., 2021), would conflict with AAVR, proposed to be the key entry receptor.

Not realized in the earlier work was: (1) that the glycans are “merely” attachment factors and not entry receptors (see above); (2) that cell surface attachment of AAV is dominated by promiscuous interactions with surface glycans and is barely affected with knock-out of AAVR (Pillay et al., 2016); and (3) in such AAVR^KO^ cell lines, productive transduction is abrogated, while strong surface attachment is maintained. Thus, while it might not have been evident 20 years ago, cell attachment data should not be interpreted as a proxy for cell entry.

Like the analysis above of glycan attachment, we can compare antibody epitopes with the structures of bound AAVR domains to examine the potential for competitive interference. Cryo-EM structures have shown that PKD2 is similarly bound by AAV1, AAV2, and AAV9 (Meyer et al., 2019; Zhang et al., 2019; Xu et al., 2022), while those closely related (AAV6 & AAV8) have been docked homologously. AAV5 and goat AAVGo.1 constitute a distinct family in which it is PKD2 that is bound strongly, and at a distinct site (Zhang et al., 2019; Silveria et al., 2020; Large et al., 2022). If we imagine that, in most cases, an antibody would extend approximately radially from the epitope (roughly parallel to the symmetry axes in Figure 5), we see that, in almost all cases, the antibody would conflict with the receptor domain. The extent of conflict ranges from severe (panels D–F, H–K) to modest (panels A–C). For panels G & L, there is not direct overlap, but with the bulk mass of antibody extending from the viral surface and the adjacent domains of AAVR connecting at the C- and N-termini, there would very likely be interference.

We do not know that the observed conflicts constitute the mechanism of neutralization. This will depend on unknown relative binding constants, concentrations of antibody, and the number of symmetry-equivalent sites on the virus that must be antibody-bound to inhibit entry. In many cases, epitopes are close to both AAVR and glycan-binding sites. [The receptor for the related simian viruses, AAV4 and AAVrh32.33, is not AAVR (Dudek et al., 2018; 2020b) but remains unidentified, so we cannot yet address antibody interference with a different AAV entry receptor. A single mAb complex, AAV4-ADK4, has been characterized at ~20 Å resolution, with ADK4 bound on the 2-fold side of the spike, like the AAV1-E4E complex (Figure 5C), and occluding the SIA attachment site (Shen et al., 2013; Emmanuel et al., 2021)]. In the complexes of the other serotypes that utilize AAVR, we see neither the complete AAVR nor glycan. The overlap with bound mAb might be more extensive than immediately apparent from structural studies, particularly when monosaccharides are used as a proxy for polysaccharide attachment. However, it is striking that in all cases characterized to date, epitopes look close enough to interfere with AAVR-binding. In several cases, antibody binding to the epitope would occlude both glycan and AAVR, so inhibition of both attachment and entry may be possible for some antibodies. For the AAV5 antibodies visualized, conflict with the AAVR receptor looks more likely than conflict with glycan attachment based on the degree of overlap between the AAV5-AAVR complex (2.5 Å) and AAV5 antibody complexes (~11 Å) (Tseng et al., 2015; Silveria et al., 2020).

Particularly intriguing is the similarity of the ADK5a and ADK5b epitopes, only the latter having been considered neutralizing (Tseng et al., 2015). With structures of the AAV5-AAVR complex (Zhang et al., 2019; Silveria et al., 2020) highlighting that both antibodies would completely occlude receptor-binding, a new attempt was made to measure competitive inhibition through ELISA (Silveria et al., 2020). Now, it was seen that both antibodies inhibited receptor-binding *et* vice versa, albeit at different concentrations that had not earlier been explored (Silveria et al., 2020).

## 7. Discussion

Much of the recent activity in AAV structural virology has focused on understanding its interactions with the newly discovered AAVR membrane protein receptor that is essential for transduction of all serotypes (except the AAV4-like clade) (Pillay et al., 2016; Dudek et al., 2018). Unanticipated has been the impact of receptor structure studies (Meyer et al., 2019; Zhang et al., 2019; Silveria et al., 2020; Hu et al., 2022; Large et al., 2022; Xu et al., 2022) on the understanding of AAV antibody neutralization that is important in the development of efficient gene delivery vectors. These unexpected insights come from the comparative analysis presented here of receptor-complex structures and earlier structures of monoclonal antibody complexes (Gurda et al., 2012; McCraw et al., 2012; Gurda et al., 2013; Tseng et al., 2015; Giles et al., 2018; Jose et al., 2019). The structures re-open the possibility that antibody binding could be interfering with AAVR-mediated cell entry, in addition to or instead of interfering with the interactions of glycans, once considered primary receptors, but now demoted to attachment factors. This is not to propose that inhibition of receptor binding is the only mechanism of neutralization, because there remain examples where epitopes are also close to glycan attachment sites. Rather the juxtaposition of antibody and AAVR binding sites, together with the new understanding of glycans as attachment factors, argue for more extensive experimental testing.

It should be emphasized that the inhibition of receptor-attachment is more of a plausible hypothesis than tested fact. There has been just one experimental test. Similar overlap of AAVR PKD1 with the ADK5a and ADK5b epitopes seemed to be at variance with previously reported neutralization activity. Reevaluation of competitive binding by ELISA has now shown that ADK5a and ADK5b are qualitatively similar in their ability to inhibit binding to AAVR, noting that such experiments fall far short of establishing that such inhibition is relevant to AAV neutralization *in vivo* (Silveria et al., 2020). Thus, comparative analysis of the available structures is a positive step forward, but much work remains.

In equating inhibition of AAVR-interactions to interference with cell entry, there is an implicit assumption that the AAV-AAVR interactions occur at the cell surface. It has been shown that AAVR is preferentially located in the peri-nuclear TGN, but that it is thought to cycle transiently to the cell membrane, in its native function as a presumptive orphan receptor (Pillay et al., 2016). Given that transduction can be inhibited by addition of anti-AAVR antibodies (Pillay et al., 2016), it is likely that the encounter occurs at the cell surface (rather than later during endocytosis). However, it has not yet been possible to demonstrate this definitively, because attachment of AAV to the cell surface is dominated by glycan interactions that would swamp detection of AAVR-bound AAV at the cell surface.

Even if inhibition of AAVR-mediated entry does not prove to be a pervasive neutralization mechanism, there are important consequences of the juxtaposition of the antibody and receptor binding sites. Vectors engineered with mutations to evade recognition by neutralizing antibodies will only be viable if the receptor-virus interface, needed for entry, is not disrupted. This will constrain the design of such vectors, limiting mutation of the epitope to regions outside the AAVR-binding site.

Our comparative analysis has been leading to reevaluation of the interaction between antibodies and receptors. It is to be emphasized that the historical virological data are sound, but that prior conclusions extended the interpretation by incorporating, as assumptions, common wisdom of the time. With hindsight, key assumptions, such as the role of glycans as primary receptors, are suspect. We cannot take the conclusions of some of the literature *prima facie*, but instead need to look back at what these data tell us with a current understanding of AAV biology.

## Data availability statement

Publicly available datasets were analyzed in this study. These data can be found at: Protein Data Bank (https://www.rcsb.org/).

## Author contributions

EL prepared the first draft, and edited subsequent manuscript versions. MC was responsible for conceptual design, funding, comparative and interpretative analysis of results and manuscript revision. All authors contributed to the article and approved the submitted version.

## Funding

This work was supported by a grant from the National Institutes of Health, R35GM122564 to MC.

## Conflict of interest

The authors declare that the research was conducted in the absence of any commercial or financial relationships that could be construed as a potential conflict of interest.

## Publisher’s note

All claims expressed in this article are solely those of the authors and do not necessarily represent those of their affiliated organizations, or those of the publisher, the editors and the reviewers. Any product that may be evaluated in this article, or claim that may be made by its manufacturer, is not guaranteed or endorsed by the publisher.
